# Prognostic Significance of the CXCLs and Its Impact on the Immune Microenvironment in Ovarian Cancer

**DOI:** 10.1155/2023/5223657

**Published:** 2023-02-06

**Authors:** Cairu Gu, Xifeng Xiong, Wei Liu

**Affiliations:** ^1^Department of Gynecology, Guangzhou Red Cross Hospital, Guangzhou Red Cross Hospital of Jinan University, Guangzhou, Guangdong 510220, China; ^2^Guangzhou Institute of Traumatic Surgery, Guangzhou Red Cross Hospital, Guangzhou Red Cross Hospital of Jinan University, Guangzhou, Guangdong 510220, China; ^3^Department of Breast, Guangzhou Red Cross Hospital, Guangzhou Red Cross Hospital of Jinan University, Guangzhou, Guangdong 510220, China

## Abstract

The chemokine (C-X-C motif) ligand (CXCL) family in tumor tissue is closely related to tumor growth, metastasis, and survival. However, the differential expression profile and prognostic value of the CXCLs in ovarian cancer (OC) have not been elucidated. Therefore, we studied the expression levels and mutations of CXCLs in OC patient in TCGA and various public databases. The expression differences of CXCLs in OC cancer tissues and normal tissues were compared through the Gene Expression Profiling Interactive Analysis (GEPIA) database. The effect of CXCLs on OC prognosis was analyzed using the Kaplan-Meier curves in GEPIA database. The impact of CXCLs on immune infiltration and clinicopathological outcomes in OC was assessed using the TIMER algorithm. Compared with normal tissues, we found that eight CXCLs were significantly differentially expressed in OC. The expression levels of CXCL9 (*P* = 0.0201), CXCL11 (*P* = 0.0385), and CXCL13 (*P* = 0.0288) were significantly associated with tumor stage. CXCL13 was the only gene that significantly affected both disease-free survival (DFS) and overall survival (OS) in OC, and higher CXCL13 transcript levels implied longer DFS and OS. Although there was no significant impact on DFS, CXCL10 (*P* = 0.0079) and CXCL11 (*P* = 0.0011) expression levels had a significant effect on OS in OC. At the same time, CXCLs were significantly associated with several immune-infiltrating cells in OC tissues. The CXCLs were significantly associated with one or more immune-infiltrating cells in OC tissue. CXCL13 was differentially expressed in OC and significantly affected the prognosis of patients and was a potential marker of OC prognosis.

## 1. Introduction

Ovarian cancer (OC) is the most common type of cancer in the female reproductive system and the fifth most common cause of cancer-related death in women worldwide [1–3]. Although the survival rate of early-stage OC is higher than before, the recurrence, metastasis, and drug resistance of OC patients are inevitable [4]. The development of more therapeutic targets is the key point to improve the survival rate of OC patients. The pathogenesis of OC is very complex, involving multiple genes and multiple molecular mechanisms. Therefore, identifying more OC molecular markers is expected to develop more therapeutic targets.

The tumor microenvironment (TME) plays an important role in tumorigenesis [5–7]. The TME consists of tumor cells and nontumor cells, such as B cells, CD4^+^T cells, CD8^+^T cells, macrophages, neutrophils, and dendritic cells [8]. These tumor-associated immune cells are known to have antitumor or protumor functions. The infiltration of these nontumor cells has been shown to modulate tumor progression and tumor therapy sensitivity. The development and progression of cancer is influenced by its surrounding tumor microenvironment. Indeed, cancer cells can functionally regulate their tumor microenvironment through the secretion of various cytokines, chemokines, and other factors [9]. This leads to alterations in peritumor cells that allow them to play a key role in tumor survival and progression. Immune cells are an important component of the TME and are critically involved in this process. There is growing evidence that the presence of innate immune cells (macrophages, neutrophils, dendritic cells, etc.) as well as adaptive immune cells (T and B cells) in the tumor microenvironment (TME) contributes to tumor progression [9]. The cross-talk between cancer cells and these immune cells ultimately leads to an environment that promotes tumor growth and metastasis [9].

The diagnosis and treatment of ovarian cancer have made great progress in recent years, such as the emergence of new materials [10–12], new drugs [13, 14], and new molecular markers or targets [15]. The heterogeneity of tumor is not only determined by the characteristics of tumor cells themselves but also affected by the characteristics of tumor immune microenvironment to a large extent. Chemokines play an important role in tumor immune microenvironment. These chemokines can be produced by cells in the tumor microenvironment and lead to the recruitment of immune cells from the circulation to the tumor site. The cysteine-X (any amino acid)-cysteine motif ligand (CXCL) family is such a group of chemokines. We hypothesized that the abundance of these CXCLs is related to the immune microenvironment to a certain extent, thereby affecting tumor heterogeneity and patient prognosis. Therefore, the biological significance of this study is to explore the relationship between CXCLs and clinicopathological characteristics and survival of patients with ovarian cancer in view of the correlation between CXCLs and tumor microenvironment.

The CXCL family plays an important role in inflammation [16]. More and more studies have shown that CXCL may directly affect tumor growth, invasion, and metastasis through the interaction between tumor cells and stromal cells. Aref et al. [17] found circulating CXCL13 as a biomarker of chronic lymphocytic leukemia severity. Wang et al. [5] found that the CXCLs were involved in the regulation of the immunosuppressive microenvironment and chemoresistance in glioma. Gu [18] et al. found that CXC1/2/8 were significantly associated with tumor characteristics and survival in patients with non-small-cell lung cancer. Lin et al. [19] found that CXCL2/10/12/14 were prognostic biomarkers in hepatocellular carcinoma and were associated with immune infiltration in hepatocellular carcinoma. Park et al. [20] found that epithelial stromal communication through CXCL1-CXCR2 interaction stimulated the growth of ovarian cancer cells through p38 activation. Snail recruit myeloid-derived suppressor cells by upregulating CXCL1/2 to promote ovarian cancer progression [21]. Seitz found that CXCL9 inhibited tumor growth and promoted anti PD-L1 treatment of ovarian cancer. Dangaj et al. [22] found that overexpression of CCL5 and CXCL9 was associated with CD8^+^ T cell infiltration in solid tumors. Coexpression of CCL5 and CXCL9 showed that immunoreactive tumors had prolonged survival and response to checkpoint blockade.

However, the expression of CXCLs in OC and their impact on prognosis remains unclear. Therefore, we investigated the expression and mutations of CXCLs in ovarian patient data in The Cancer Genome Atlas (TCGA) and various public databases. Using a variety of analytical methods, we analyzed genomic changes and functional networks associated with the expression of CXCLs in OC. Thus, our findings may reveal new molecular targets for OC diagnosis and prognosis.

## 2. Materials and Methods

### 2.1. Analysis of Differential Expression of CXCLs in OC

We investigated the expression of the CXCLs in OC and normal tissues through the Gene Expression Profiling Interactive Analysis (GEPIA) database (http://gepia.cancer-pku.cn/index.htm) [23]. GEPIA included TCGA and Genotype-Tissue Expression (GTEx) RNA-sequencing expression data, which included a total of 426 OC tumor samples and 88 normal tissues. The “single gene” module of GEPIA was used to study the mRNA expression level of the CXCLs in OC tissues and normal tissues. Multiple gene comparison analysis was then performed using the “Multiple genes comparison” module from GEPIA and the “ovarian cancer” dataset. The Kaplan-Meier curves were used to study the survival outcome, including disease-free survival (DFS) and overall survival (OS). OC patients were divided into high and low CXCL groups according to the median transcripts per million (TPM) expressions. The methods used in this study refer to Lu et al.'s previously published paper [12]. GSE66957 dataset contains mRNA expression profiles of 57 ovarian cancer and 12 normal ovarian samples. The expression of CXCLs in OC in GSE66957 dataset was carried out through easyGEO database (https://tau.cmmt.ubc.ca/eVITTA/easyGEO/).

### 2.2. Genetic Alteration Analysis of CXCLs in OC Patients

cBioPortal (http://www.cbioportal.org) is an online data analysis website with easy access to data that enables visualization of multidimensional analysis [24]. Via the TCGA database, we assessed genomic alterations associated with the CXCLs in OC to explore their potential roles in tumorigenesis.

### 2.3. Protein-Protein Interaction (PPI) Network Analysis of the CXCLs

GeneMANIA (http://www.genemania.org) is a platform that can be used for gene function prediction [25]. Given a query gene, GeneMANIA finds genes that may share function with it based on the gene's interactions with it. STRING (https://string-db.org) is a web platform for studying functional protein interaction networks [26]. Predicted protein-protein interactions (PPIs) were generated using GeneMANIA and STRING.

### 2.4. Gene Ontology and Pathway Enrichment Analysis

WebGestalt (http://www.webgestalt.org/option.php) is an online and powerful tool for enrichment analysis [27]. In current study, we used “Overrepresentation Analysis” (ORA) as our method of interest and “gene ontology and Pathways” as a functional database to validate the enrichment of the CXCLs.

### 2.5. Relationship between CXCLs and Immune Infiltration in OC Tumor Microenvironment

TIMER is an easily available online tool for the systematic analysis of immune infiltration in multiple tumors [28]. In this study, we used the TIMER algorithm to estimate the immune properties of tumors, and the “genes” module was used to assess the relationship between CXCLs and immune cell infiltration (B cells, CD4^+^ T cells, CD8^+^ T cells, macrophages, neutrophils, and dendritic cells).

### 2.6. Statistical Analysis

Statistical analysis was performed by the built-in software of each data. Differences between cancer tissues and normal tissues were analyzed by *t*-test, and Spearman's correlation coefficient was used to analyze the correlation. Using the median of TPM as the cut-off value, they were divided into high CXCL group and low CXCL group. The Kaplan-Meier curves were used to show DFS and OS, and the log-rank test was used to compare differences between groups. Significant differences were defined as when *P* < 0.05.

## 3. Results

### 3.1. Expression Profiles of CXCLs in OC Tissues

**Figure 1:**
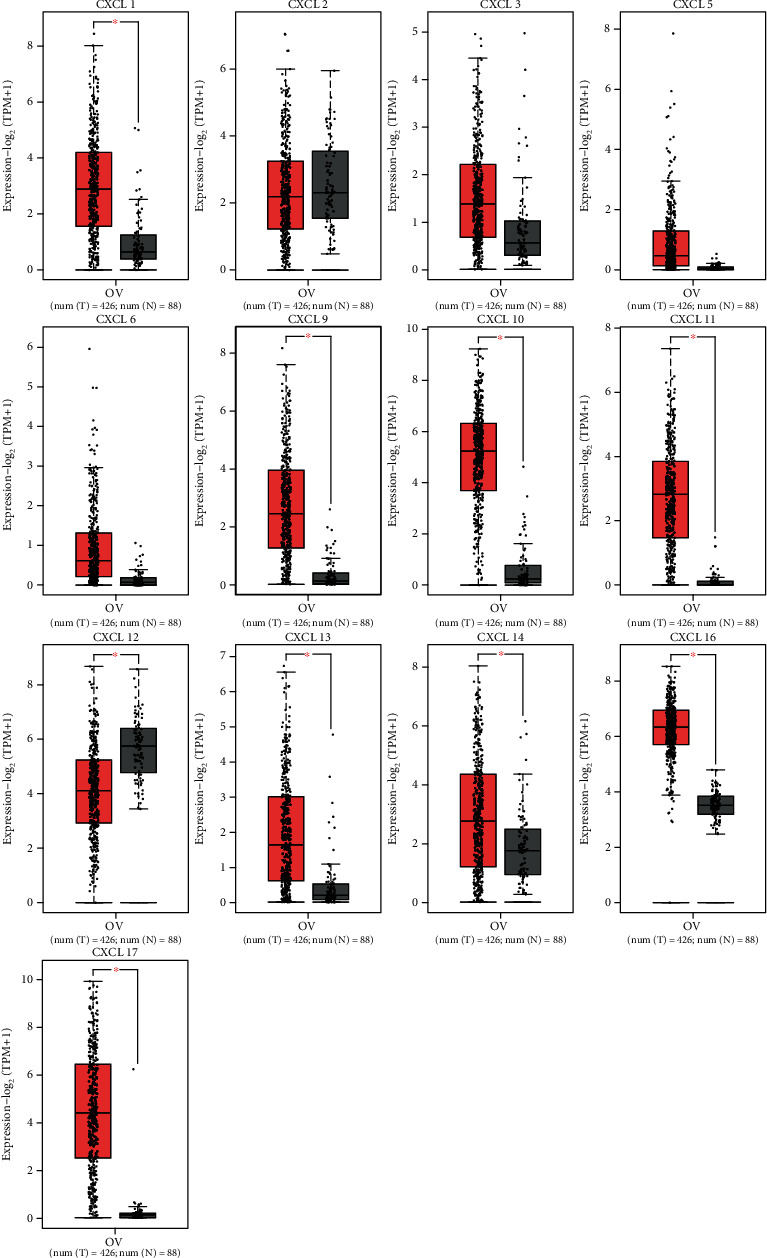
Expression levels of the CXCLs in OC and normal tissues. The expression status of CXCLs in OC (red box plots) and normal tissues (gray box plots) was analyzed by TIMER2. ^∗^*P* < 0.05. Abbreviations: OC: ovarian cancer; CXCLs: cysteine-X (any amino acid)-cysteine motif ligands.

**Figure 2:**
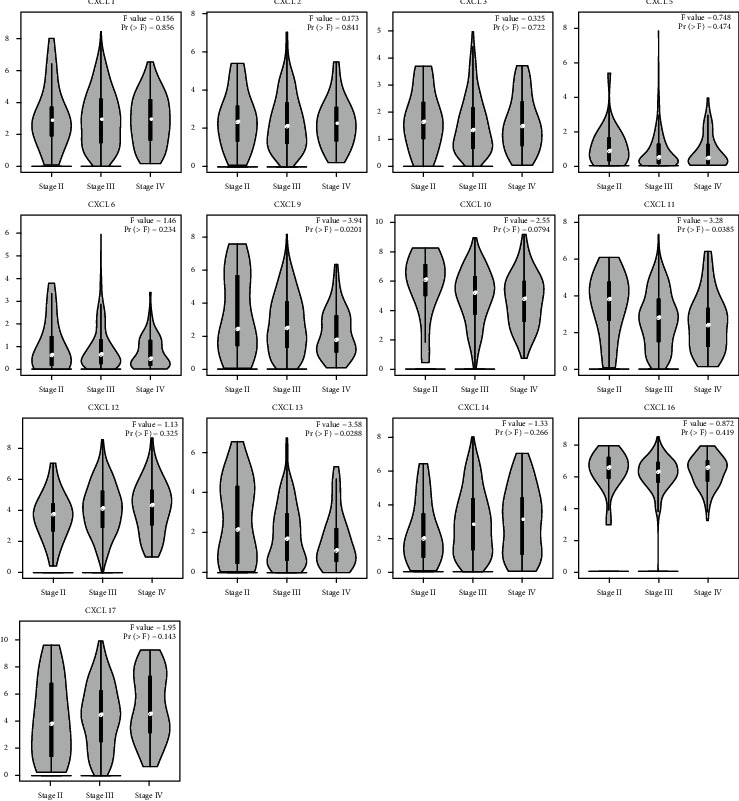
Effect of CXCL expression level on tumor stage in OC patients. ^∗^*P* < 0.05. The mRNA expression in CXCL9/11/13 was significantly related to the cancer stage of OC patients, while the mRNA expression of other CXCLs was not significantly related to this. Abbreviations: OC: ovarian cancer; CXCLs: cysteine-X (any amino acid)-cysteine motif ligands.

We identified the expression of 13 CXCLs in OC from the GEPIA database. As shown in Figure 1, data of 426 OC and 88 normal tissues were studied. The transcription level of CXCL12 in OC tissues was significantly lower than that in normal tissues, while the transcription levels of CXCL1, CXCL9, CXCL10, CXCL11, CXCL13, CXCL14, CXCL16, and CXCL17 were significantly increased in OC tissues. In addition, we found that the mRNA expression levels of CXCL1, CXCL5, CXCL6, CXCL9, CXCL10, CXCL11, CXCL13, CXCL14, and CXCL16 in OC tissues were significantly increased in GSE66957 dataset (Supplementary Figure 1). We also found that abnormal expression of CXCL9 (*P* = 0.0201), CXCL11 (*P* = 0.0385), and CXCL13 (*P* = 0.0288) was closely associated to tumor stage (Figure 2), and the expression of CXCL9, CXCL11, and CXCL13 decreased with the increase of stage.

### 3.2. Prognostic Analysis of CXCLs in OC Patients

**Figure 3:**
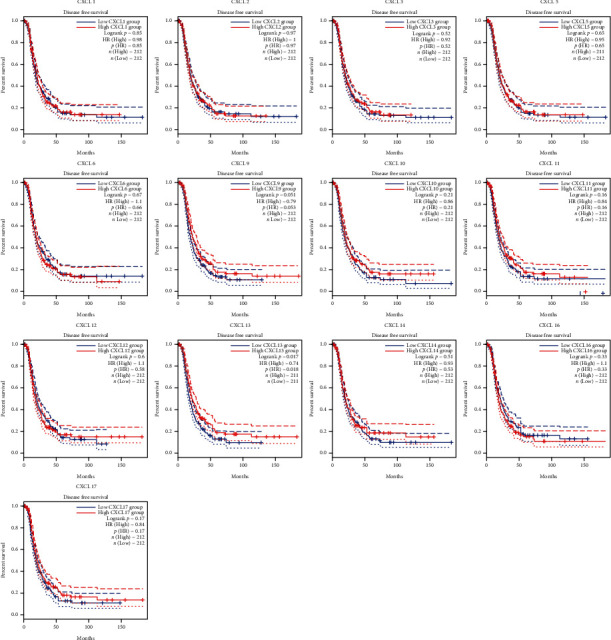
The effect of CXCLs on disease-free survival (DFS) of ovarian cancer patients. Curves were drawn comparing DFS based on high (red curve) and low (blue curve) levels of CXCL gene expression in OC. *P* value less than 0.05 is defined as statistical difference. Abbreviations: OC: ovarian cancer; CXCLs: cysteine-X (any amino acid)-cysteine motif ligands; DFS: disease-free survival.

**Figure 4:**
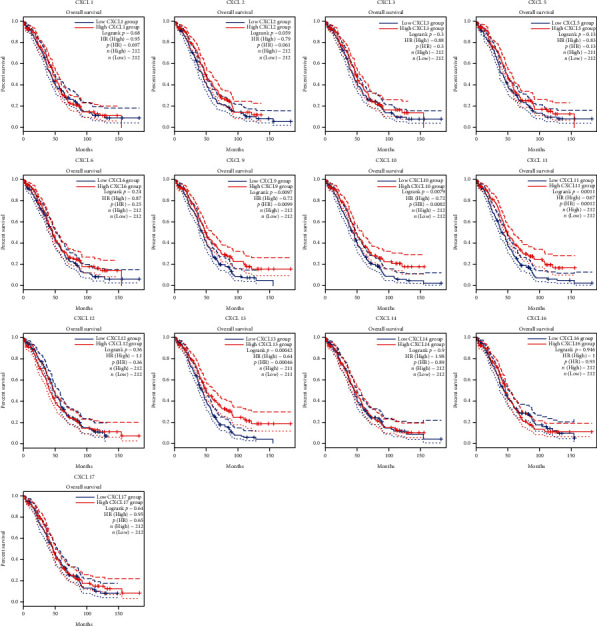
The effect of CXCLs on overall survival (OS) of OC patients. Curves were drawn comparing OS based on high (red curve) and low (blue curve) levels of CXCLs expression in OC. *P* value less than 0.05 is defined as statistical difference. Abbreviations: OC: ovarian cancer; CXCLs: cysteine-X (any amino acid)-cysteine motif ligands; OS: overall survival.

The Kaplan-Meier curves were used to describe DFS and OS of OC patients in the GEPIA dataset to clarify the independent prognostic value of CXCLs in OC. The median RNA-seq expression value was used as the cut-off value to divide into high and low expression groups. As shown in Figure 3, patients with high transcript levels of CXCL13 (*P* = 0.017) were significantly associated with longer DFS, while other CXCLs were not significantly associated with DFS in OC (Figure 3). In terms of their effect on OS, we observed that patients with high transcript levels of CXCL10 (*P* = 0.0079), CXCL11 (*P* = 0.0011), and CXCL13 (*P* = 0.00042) were significantly associated with longer OS; the remaining CXCLs were not significantly associated with OS of OC (Figure 4).

### 3.3. Genetic Alterations and PPI Network Analysis in OC Patients

**Figure 5:**
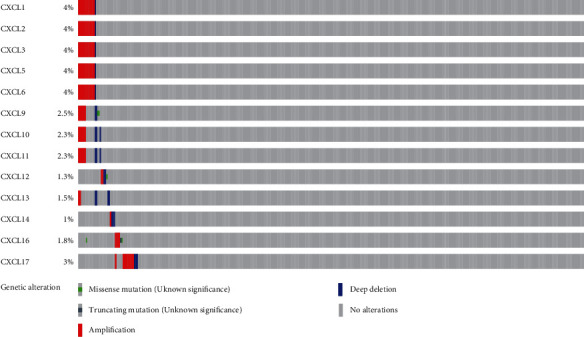
Summary of genetic alterations of CXCLs in OC. The color represents the number of examples of amplification, the blue represents deep deletion, the green represents missense mutation, and the gray represents no mutation. Abbreviations: OC: ovarian cancer; CXCLs: cysteine-X (any amino acid)-cysteine motif ligands.

To further analyze mutated genes of CXCLs in OC, 585 samples from TCGA were analyzed using the cBioPortal dataset. Genetic alterations in OC from members of the CXCLs are as follows (Figure 5): CXCL1 (4%), CXCL2 (4%), CXCL3 (4%), CXCL4 (4%), CXCL5 (4%), CXCL6 (4%), CXCL9 (2.5%), CXCL10 (2.3%), CXCL11 (2.3%), CXCL12 (1.3%), CXCL13 (1.5%), CXCL14 (1%), CXCL16 (1.8%), and CXCL17 (3%). In OC, amplification was the most common type of genetic alteration of CXCLs. GeneMANIA also generated PPIs of the CXCLs to assess their interaction (Supplementary Figure 2A). The main biological functions of the CXCLs are related to cytokine activity, chemokine receptor binding, cellular response to chemokine, response to chemokine, and granulocyte chemotaxis. Supplementary Figure 2B shows the predicted interaction network between CXCLs.

### 3.4. Gene Ontology (GO) and Kyoto Encyclopedia of Genes and Genomes (KEGG) Analyses of the CXCLs

We use the WebGestalt database to perform the GO analysis and KEGG pathway of the CXCLs. Supplementary figure 3A shows the GO analysis of CXCLs in three aspects: cellular composition (CC), biological process (BP), and molecular function (MF). Our results suggested that the CXCLs are mainly distributed in extracellular space, and their main biological functions include cell communication and response to stimuli, and their molecular functions are mainly related to protein binding (Supplementary Figure 3A). The top 6 KEGG pathways were IL-17 signaling pathway, chemokine signaling pathway, legionellosis, TNF signaling pathway, and rheumatoid arthritis. Some of the enriched pathways are closely related to the occurrence and progression of OC (Supplementary Figure 3B).

### 3.5. Correlation Analysis of CXCLs and Infiltrating Immune Cells

**Figure 6:**
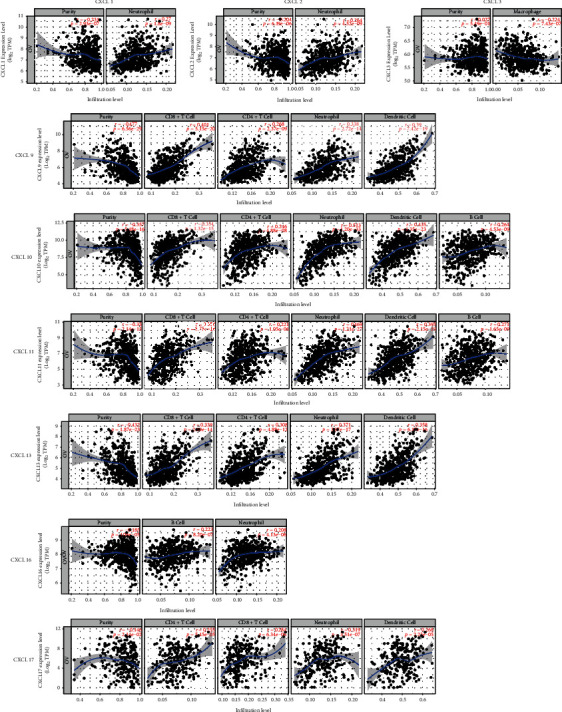
Correlation of CXCLs with 6 major types of infiltrating immune cells (B cells, CD8^+^ T cells, CD4^+^ T cells, macrophages, neutrophils, and dendritic cells). The Spearman correlation study was used in this part of the analysis. Cor refers to the correlation coefficient, whose value fluctuates from -1 to 1. The larger the absolute value of Cor is, the stronger the correlation is. Negative values represent negative correlations, while positive values represent positive correlations.

Finally, we analyzed the expression of CXCLs and infiltrating immune cells (including B cells, CD8+ T cells, CD4+ T cells, macrophages, neutrophils, and dendritic cells) using the TIMER database (Figure 6). Correlation analysis of CXCLs and infiltrating immune cells is shown in Supplementary Figure 4. The Spearman correlation study was used in this part of the analysis.

CXCL1 (*R* = 0.27, *P* = 1.80*e* − 09), CXCL2 (*R* = 0.264, *P* = 4.52*e* − 09), CXCL9 (*R* = 0.338, *P* = 2.72*e* − 14), CXCL10 (*R* = 0.513, *P* = 1.20*e* − 33), CXCL11 (*R* = 0.469, *P* = 1.21*e* − 27), CXCL13 (*R* = 0.371, *P* = 3.97*e* − 17), CXCL16 (*R* = 0.208, *P* = 4.13*e* − 06), and CXCL17 (*R* = 0.319, *P* = 3.51*e* − 07) were significantly related to neutrophils. CXCL9 (*R* = 0.401, *P* = 5.15*e* − 20), CXCL10 (*R* = 0.354, *P* = 1.32*e* − 15), CXCL11 (*R* = 0.356, *P* = 8.78*e* − 16), CXCL13 (*R* = 0.339, *P* = 2.19*e* − 14), and CXCL17 (*R* = 0.284, *P* = 6.34*e* − 6) were significantly related to CD8^+^ T cells. CXCL9 (*R* = 0.268, *P* = 2.37*e* − 9), CXCL10 (*R* = 0.246, *P* = 4.88*e* − 08), CXCL11 (*R* = 0.221, *P* = 1.05*e* − 06), and CXCL13 (*R* = 0.308, *P* = 4.88*e* − 12) were significantly related to CD4^+^ T cells. CXCL9 (*R* = 0.39, *P* = 7.42*e* − 19), CXCL10 (*R* = 0.435, *P* = 1.35*e* − 23), CXCL11 (*R* = 0.395, *P* = 2.15*e* − 19), CXCL13 (*R* = 0.358, *P* = 6.17*e* − 16), and CXCL17 (*R* = 0.268, *P* = 2.23*e* − 5) were significantly related to dendritic cells. CXCL10 (*R* = 0.264, *P* = 4.53*e* − 09), CXCL11 (*R* = 0.271, *P* = 1.65*e* − 09) and CXCL16 (*R* = 0.223, *P* = 8.20*e* − 7) were significantly related to B cells. CXCL3 expression was negatively associated macrophage infiltration (*R* = −0.224, *P* = 7.43*e* − 07).

## 4. Discussion

The roles of the CXCLs in tumorigenesis, proliferation and metastasis, drug resistance, and angiogenesis are being increasingly reported [29, 30]. In the current study, we demonstrated the abnormal expression profile of CXCLs in OC patients. Meanwhile, the expression levels of CXCL10, CXCL11, and CXCL13 were also found to significantly affect the overall survival of OC patients.

Our results showed that the transcript levels of CXCL1/9/10/11/14/16/17 were significantly upregulated, whereas the transcript expression of CXCL12 was lower in OC tissues than in normal tissues. Interestingly, we also found that the abnormal expression of CXCL9, CXCL11, and CXCL13 is significantly associated to tumor stage, indicating that these CXCLs are closely related to tumor invasion and metastasis. Interestingly, in terms of prognostic value, we found that the transcript levels of CXCL10, CXCL11, and CXCL13 significantly affected the overall survival of OC patients. However, in terms of DFS, only CXCL13 had a significant effect. Similarly, CXCL13 has been identified as a prognostic marker in various cancers [31], such as breast cancer [32], colorectal cancer [30], and clear cell renal cell carcinoma [33, 34]. Zhao et al. [35] found that CXCL13 promotes intestinal tumorigenesis by activating epithelial AKT signaling. CXCL13 is known as a B lymphocyte chemotactic agent, initially detected in the stromal cells of B cell follicles and associated with the recruitment of B and T cell subsets [36]. CXCL13 is a key molecular determinant of tertiary lymphoid structure (TLS) formation, which is an organized aggregate of T, B, and dendritic cells involved in adaptive antitumor immune responses. Consistent with this, the expression of CXCL13 was positively correlated with infiltration of B cells, CD4^+^ T cells, CD8^+^ T cells, neutrophils, and dendritic cells.

Subsequently, we further explored their molecular features in OC tissues and found that CXCLs in OC tissues had lower levels of genetic alterations. Of these, amplification is the most common type. Gene mutation plays an important role in the occurrence and development of OC, which provides an idea for the targeted therapy of OC.

Previous studies on the tumor microenvironment have confirmed that tumor-infiltrating immune cells play a regulatory role in tumor invasion and metastasis [37–39]. Our results indicated that the expression of most CXCLs in OC is were significantly correlated to one or more immune cells (including B cells, CD8^+^ T cells, CD4^+^ T cells, macrophages, neutrophils, and dendritic cells). This indicated that the interaction between the CXCLs and immune cells was broadly involved in the immune microenvironment of tumorigenesis, and some of CXCLs can be used as a molecular marker for evaluating the immune microenvironment of OC.

We acknowledged that this study also had some limitations. First, our results were mainly based on bioinformatics analysis, but we were temporarily unable to obtain information on patients' treatment regimens from bioinformatics analysis platforms, so we cannot explore whether CXCL expression has an impact on patients' treatment sensitivity. Second, our results suggest that CXCLs may be involved in regulating the immune microenvironment of OC. However, the specific details of the regulatory relationship are lacking, and the results of this study still need to be further explored by much more in vitro and in vivo experiments. Third, although CXCL13 is the only marker that has an effect on both DFS and OS of patients, this study did not deeply explore the specific role and molecular mechanism of CXCL13 in OC, and more molecular biology research is needed to further study the molecular mechanism of CXCL13.

In conclusion, we fully investigated the differential expression and prognostic significance of CXCLs in OC in this study. First, this study provides preliminary evidence for CXCL13 as a potential marker of OC prognosis. At the same time, our findings showed that CXCLs were significantly associated with several immune-infiltrating cells (including B cells, CD8^+^ T cells, CD4^+^ T cells, macrophages, neutrophils, and dendritic cells) in OC tissues. However, the data mining-based analysis results of this study still need more research to verify, including functional tests and molecular mechanisms, which will help to further clarify the regulatory role of the CXCLs in OC.

## Data Availability

All data generated or analyzed in this study are included in this article.

## Supplementary Materials

Supplementary 1 Supplementary Figure 1: the mRNA expression level of CXCLs in OC tissues in GSE66957 dataset. ^∗^*P* < 0.05. Abbreviations: OC: ovarian cancer; CXCLs: cysteine-X (any amino acid)-cysteine motif ligands.

Supplementary 2 Supplementary Figure 2: protein-protein interaction (PPI) network analysis of the CXCLs. (A) PPI network generated from GeneMANIA; (B) PPI network generated from STRING.

Supplementary 3 Supplementary Figure 3: Gene Ontology (GO) and Kyoto Encyclopedia of Genes and Genomes (KEGG) analyses of the CXCLs. (A) Bar charts of GO analysis. (B) Bar charts of KEGG pathway analysis.

Supplementary 4 Supplementary Figure 4: Correlation analysis of CXCLs and infiltrating immune cells. The results showed that the expression of CXCL1 was positively associated with the infiltration of macrophages (*R* = 0.124, *P* = 6.54*e* − 03), neutrophils (*R* = 0.27, *P* = 1.80*e* − 09), and dendritic cells (*R* = 0.10, *P* = 2.87*e* − 02). CXCL2 expression was positively associated with CD4^+^ T cell (*R* = 0.107, *P* = 1.94*e* − 2), neutrophil (*R* = 0.264, *P* = 4.52*e* − 09), and dendritic cell infiltration (*R* = 0.129, *P* = 4.52*e* − 03) but negatively associated with macrophage infiltration (*R* = −0.172, *P* = 1.58*e* − 04; Figure 6(b)). CXCL3 expression was negatively associated with CD8^+^ T cells (*R* = 0.096, *P* = 1.16*e* − 2), B cells (*R* = −0.115, *P* = 1.16*e* − 02), and macrophage infiltration (*R* = −0.224, *P* = 7.43*e* − 07). CXCL5 expression was negatively associated with CD8^+^ T cells (*R* = −0.092, *P* = 4.36*e* − 2) and macrophage infiltration (*R* = −0.098, *P* = 3.22*e* − 02) but was significantly positively correlated with CD4^+^ T cells (*R* = 0.116, *P* = 1.07*e* − 2) and neutrophils (*R* = −0.137, *P* = 2.63*e* − 3). CXCL6 expression was positively correlated with CD4^+^T cells (*R* = 0.09, *P* = 4.87*e* − 2) and neutrophils (*R* = −0.13, *P* = 4.39*e* − 3). CXCL9 expression was positively correlated with all immune cell types except macrophages (B cells, CD8^+^ T cells, CD4^+^ T cells, neutrophils, and dendritic cells; all *P* < 0.05). The expression of CXCL10 and CXCL11 was positively correlated with the infiltration of B cells, CD8^+^ T cells, CD4^+^ T cells, macrophages, neutrophils, and dendritic cells (all *P* < 0.05). CXCL12 expression was positively correlated with infiltration of CD4^+^ T cells (*R* = 0.131, *P* = 4.15*e* − 3) and macrophages (*R* = 0.181, *P* = 6.93*e* − 5) but negatively correlated with infiltration of B cells (*R* = −0.112, *P* = 1.39*e* − 2). The expression of CXCL13 and CXCL16 was positively correlated with other immune cells except macrophages. The expression of CXCL14 was not significantly associated with the infiltration of all six immune cells. CXCL17 expression was positively correlated with B cells (*R* = 0.255, *P* = 5.45*e* − 5), CD8^+^ T cells (*R* = 0.284, *P* = 6.34*e* − 6), neutrophils (*R* = 0.319, *P* = 3.31*e* − 7), and infiltration of dendritic cells (*R* = 0.268, *P* = 2.23*e* − 5).
