# Supplementary figures and images for: Prognostic Significance of the CXCLs and Its Impact on the Immune Microenvironment in Ovarian Cancer
Source: Dis Markers. 2023 Feb 6;2023:5223657. doi: 10.1155/2023/5223657 (PMC9926335; doi:10.1155/2023/5223657)

CXCL1

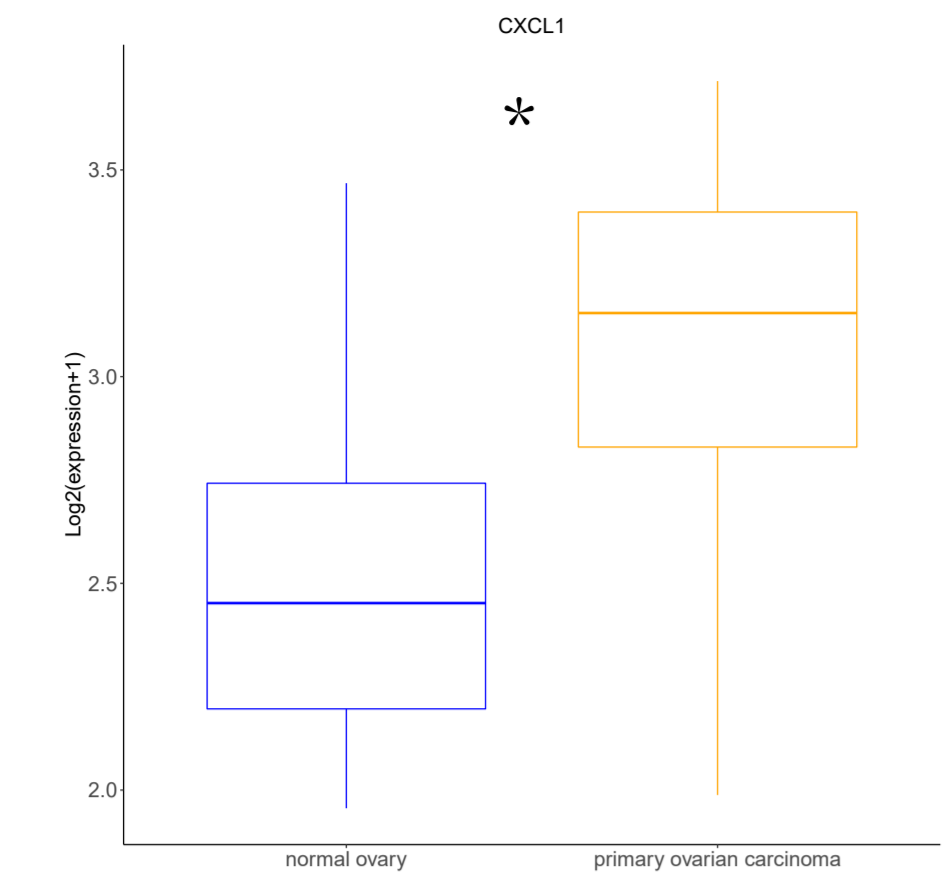

CXCL2

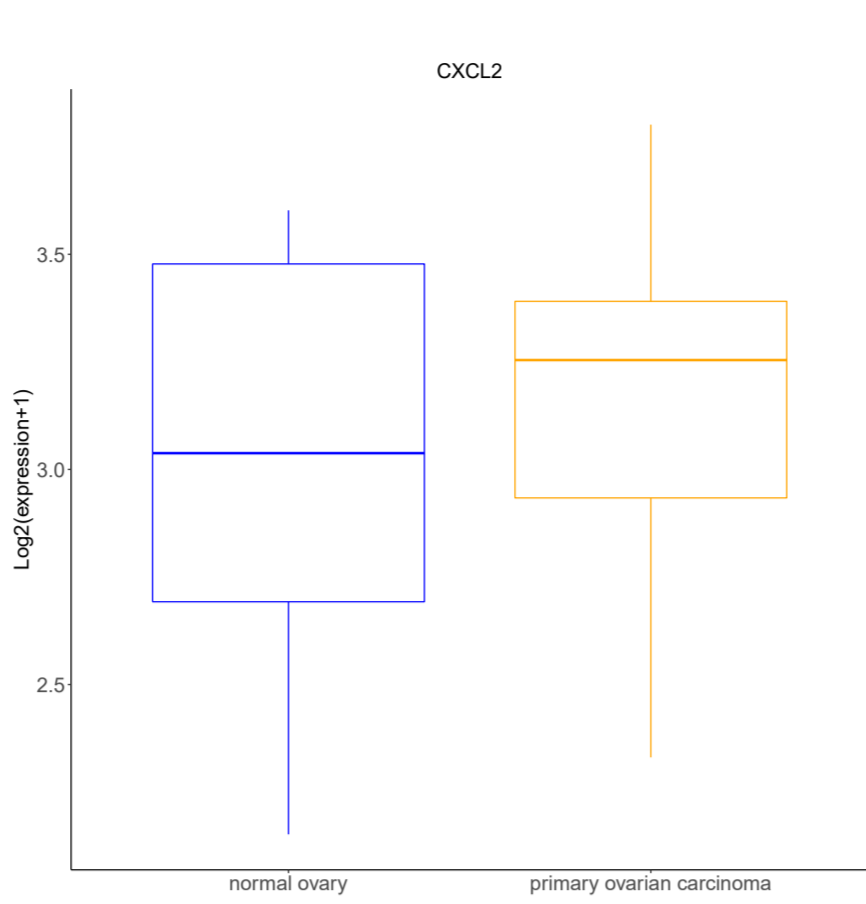

CXCL3

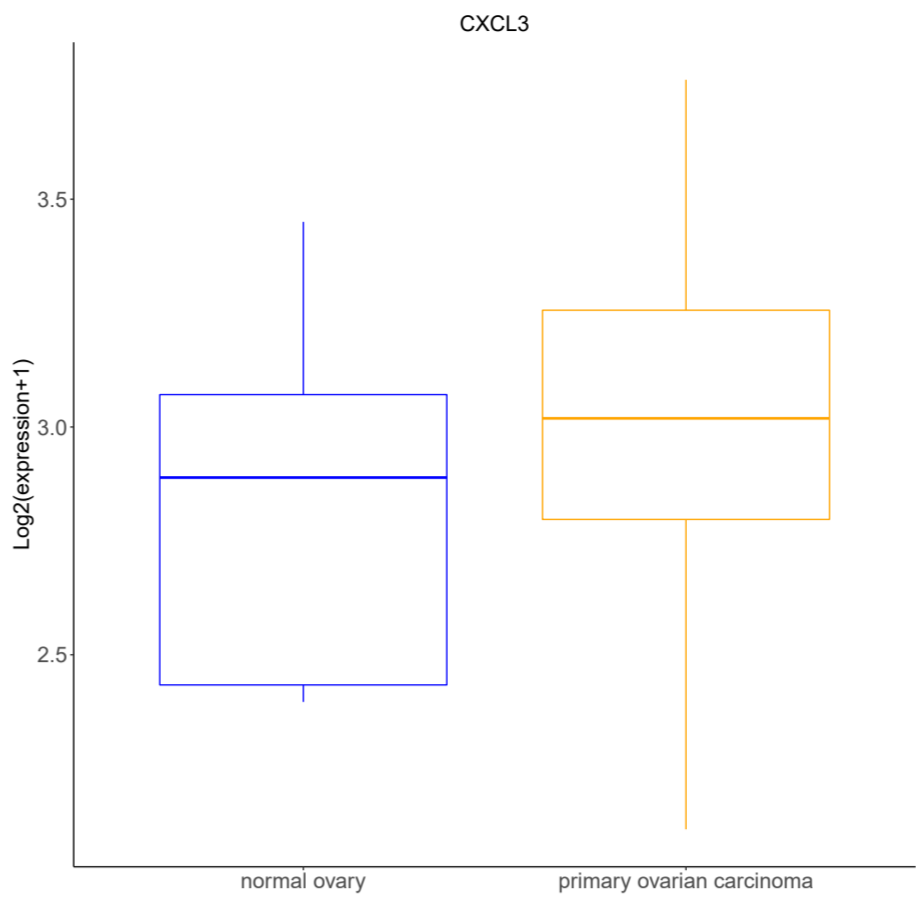

CXCL5

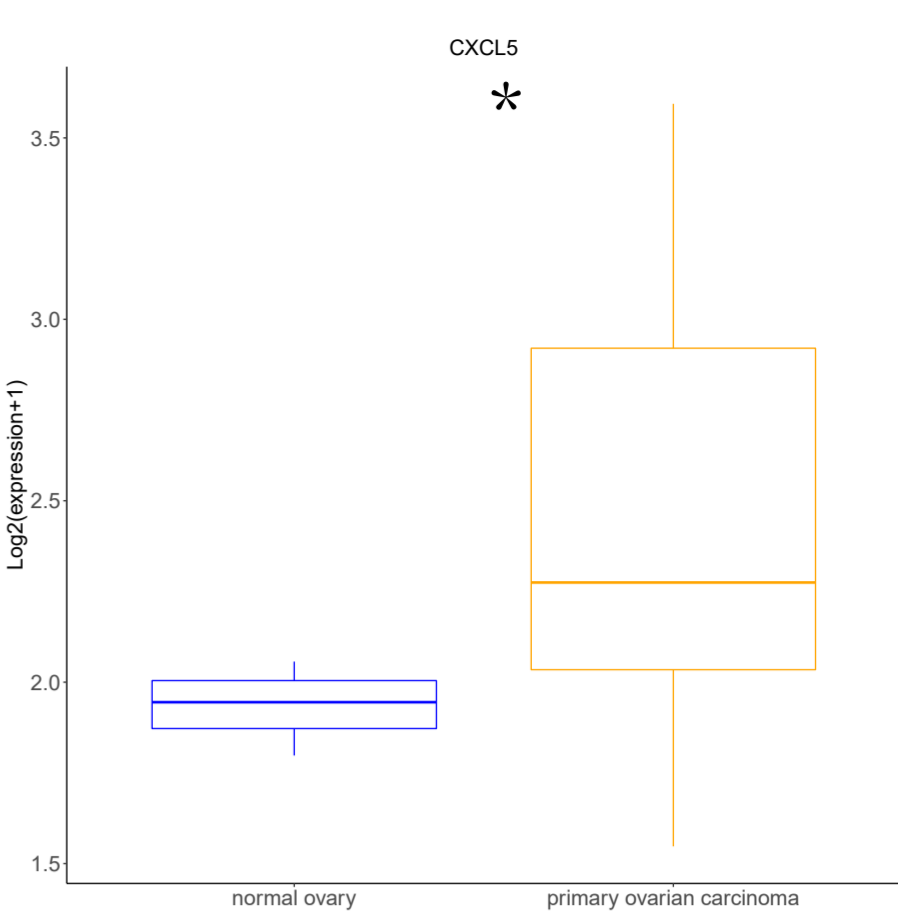

CXCL6

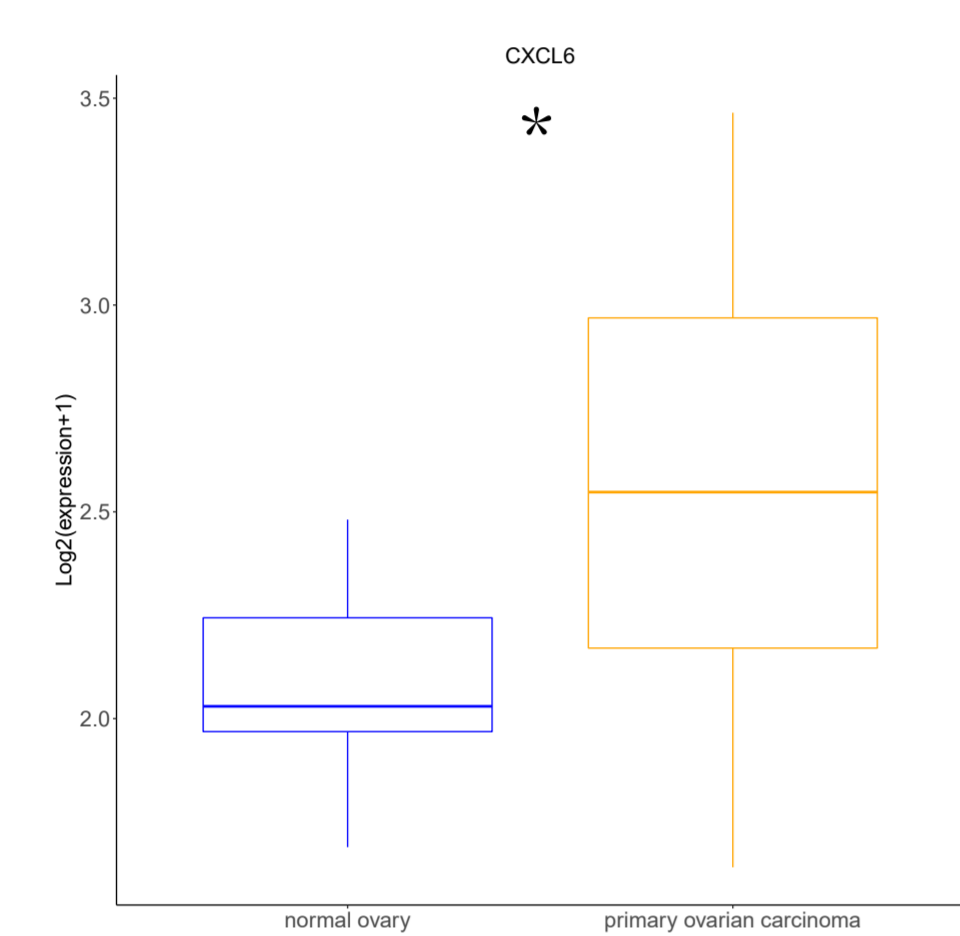

CXCL9

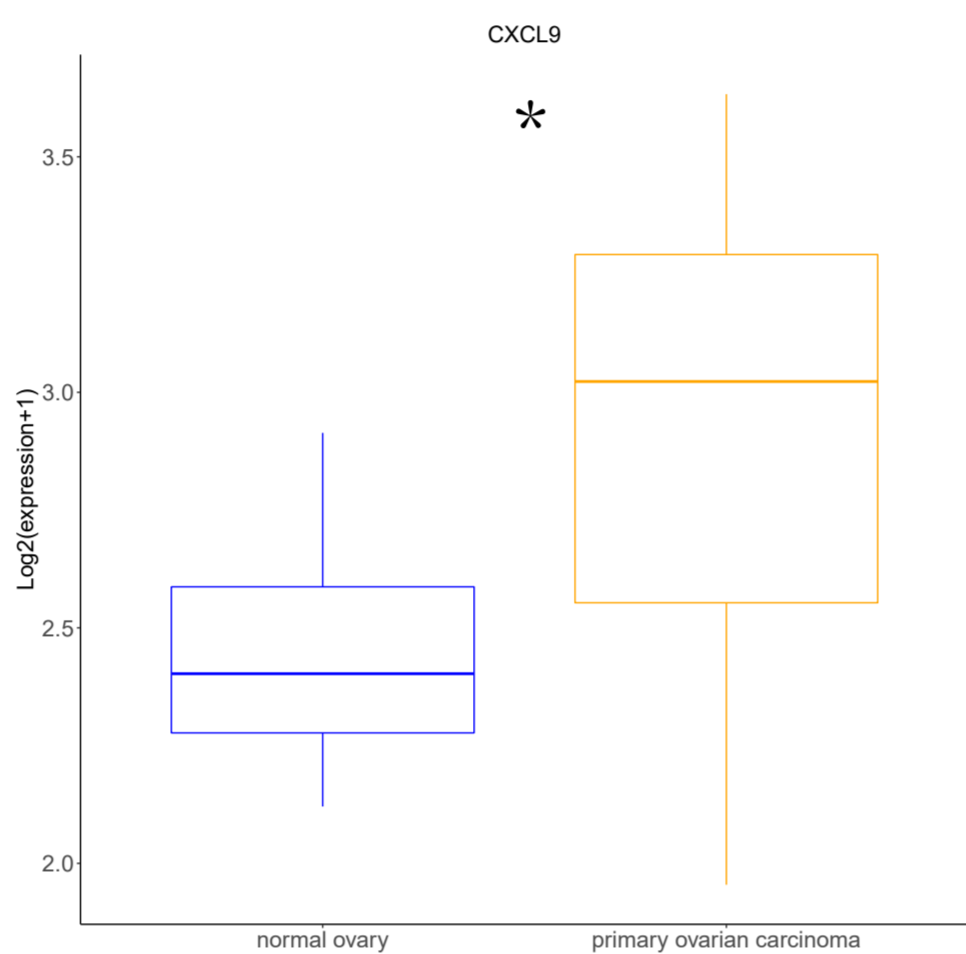

CXCL10

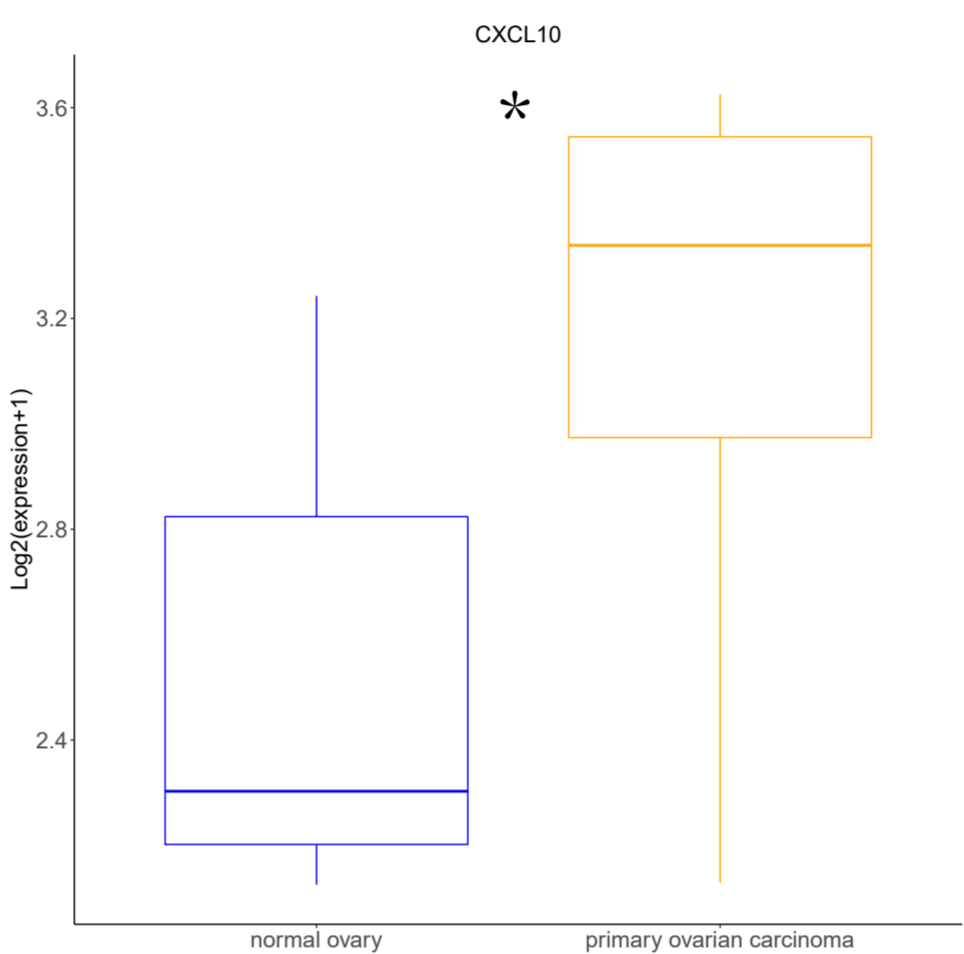

CXCL11

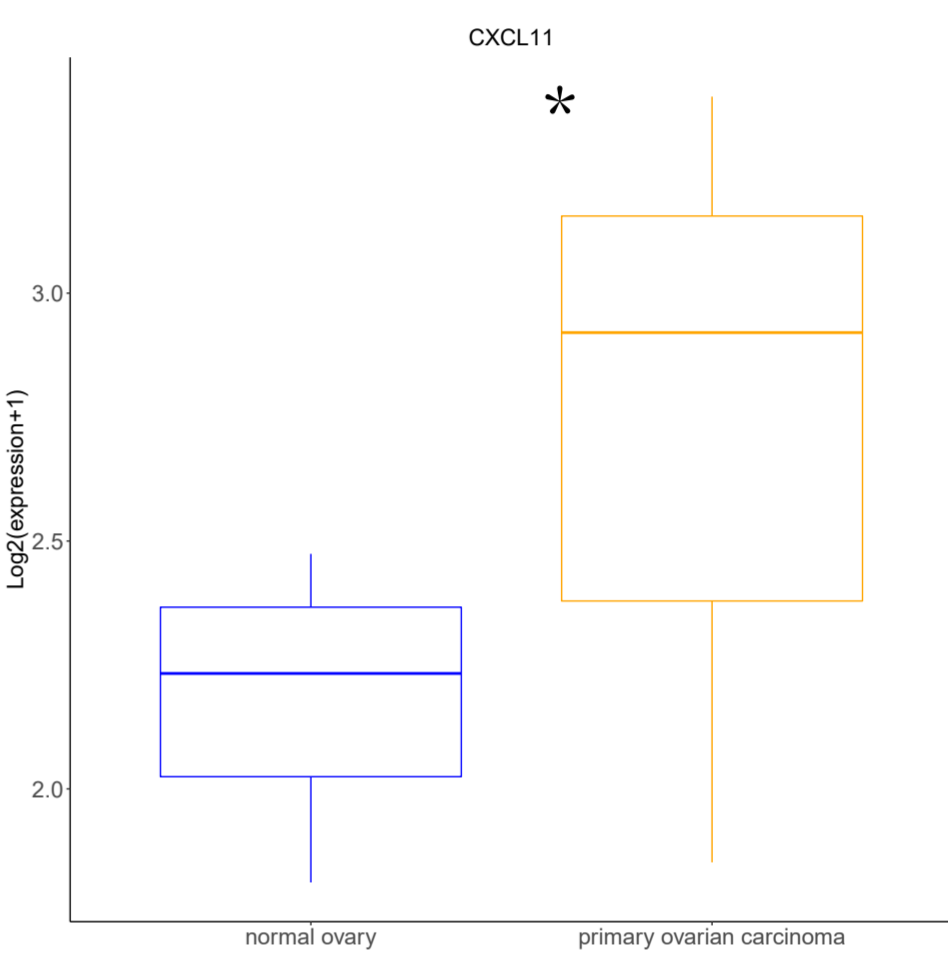

CXCL12

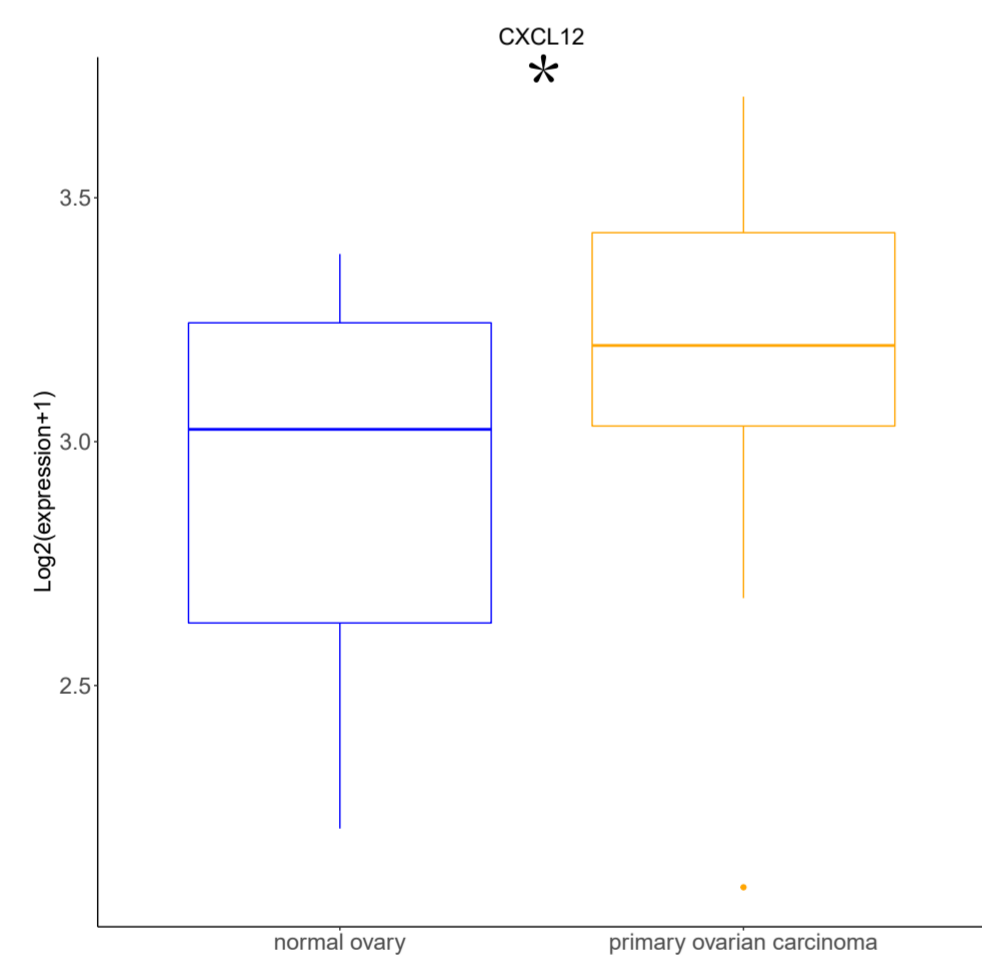

CXCL13

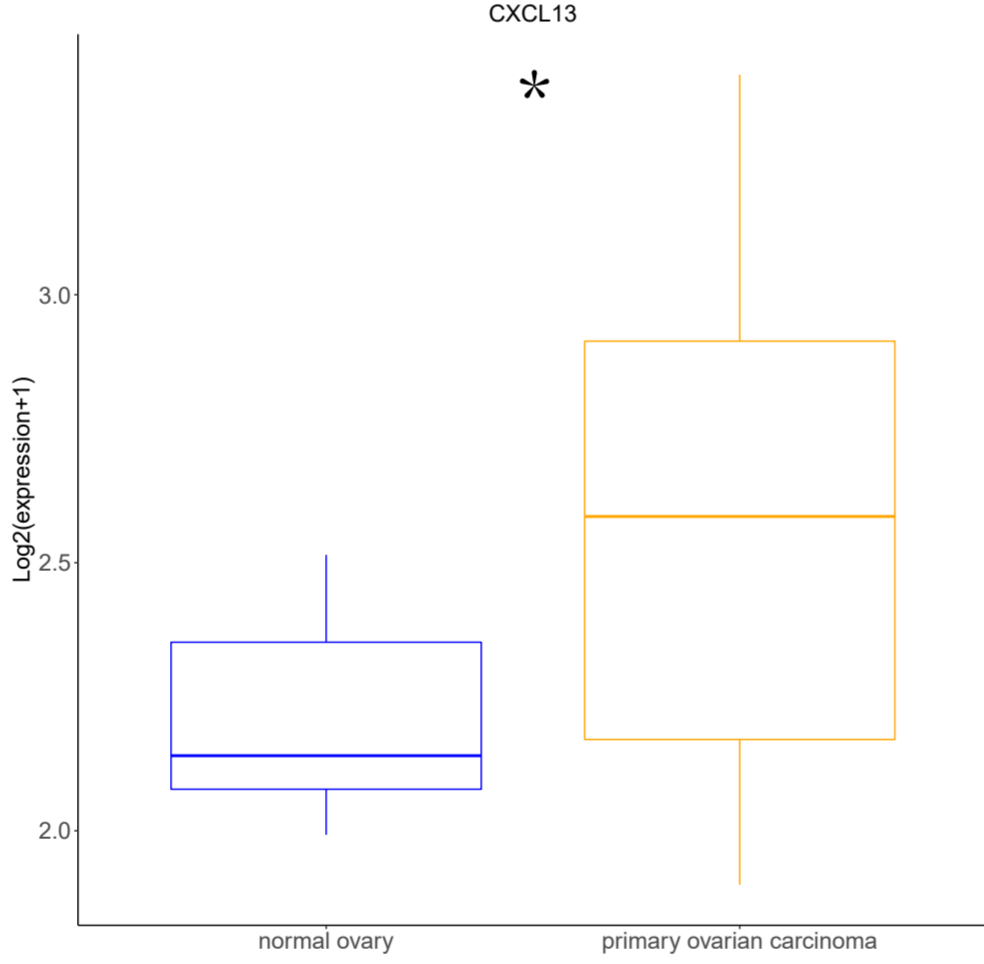

CXCL14

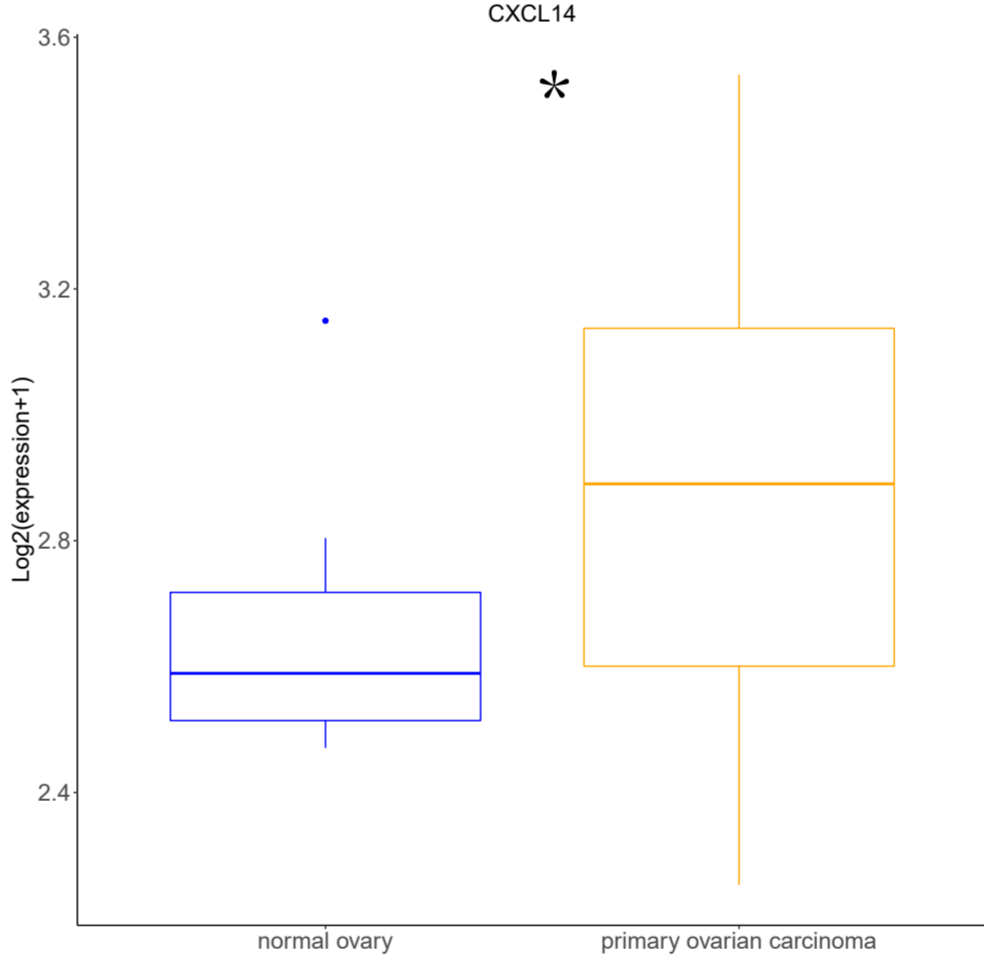

CXCL16

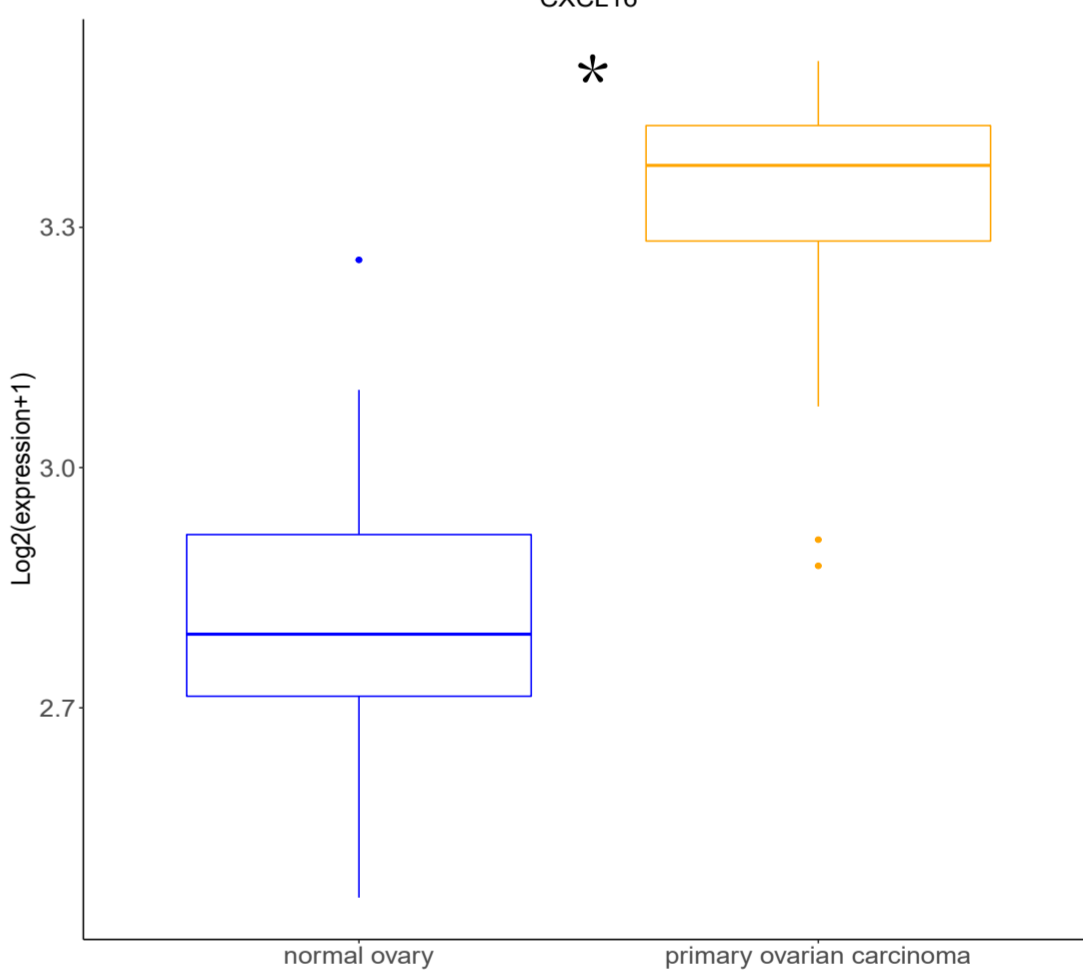

CXCL17

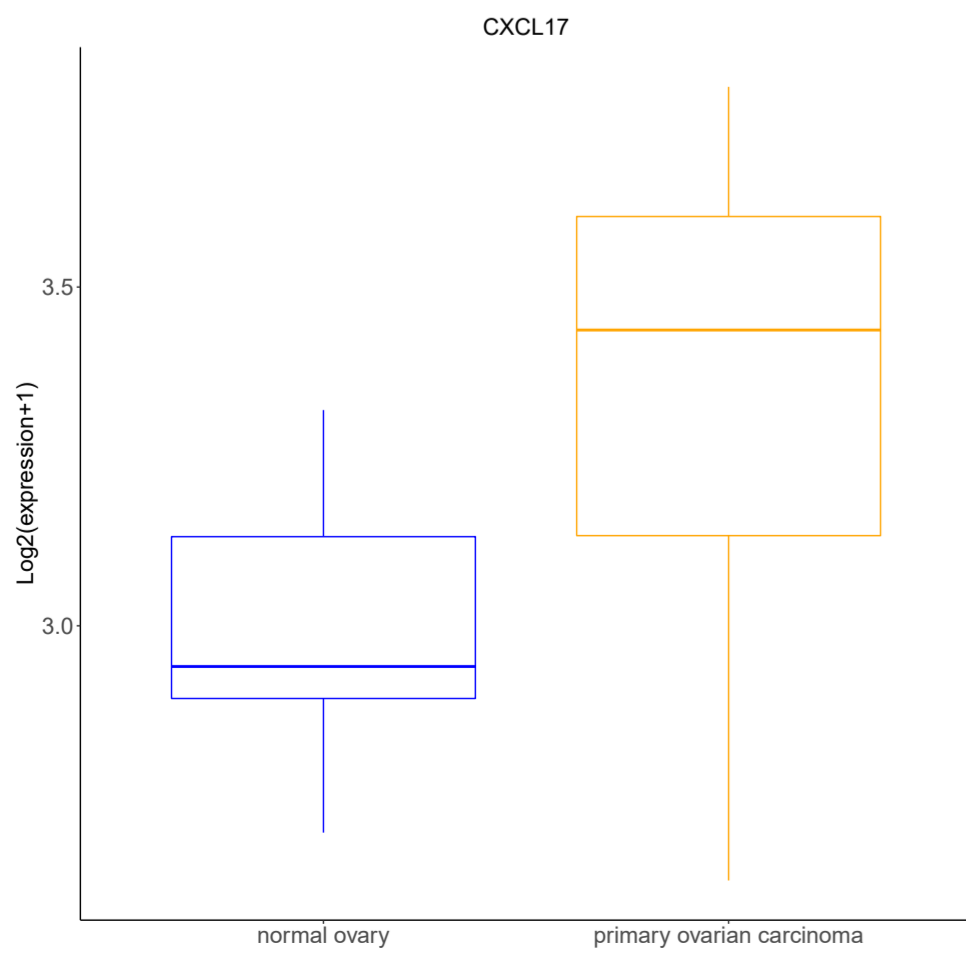

Supplement: Supplementary 1 — Supplementary Figure 1: the mRNA expression level of CXCLs in OC tissues in GSE66957 dataset. ∗P < 0.05. Abbreviations: OC: ovarian cancer; CXCLs: cysteine-X (any amino acid)-cysteine motif ligands. [file 5223657.f1.pdf]

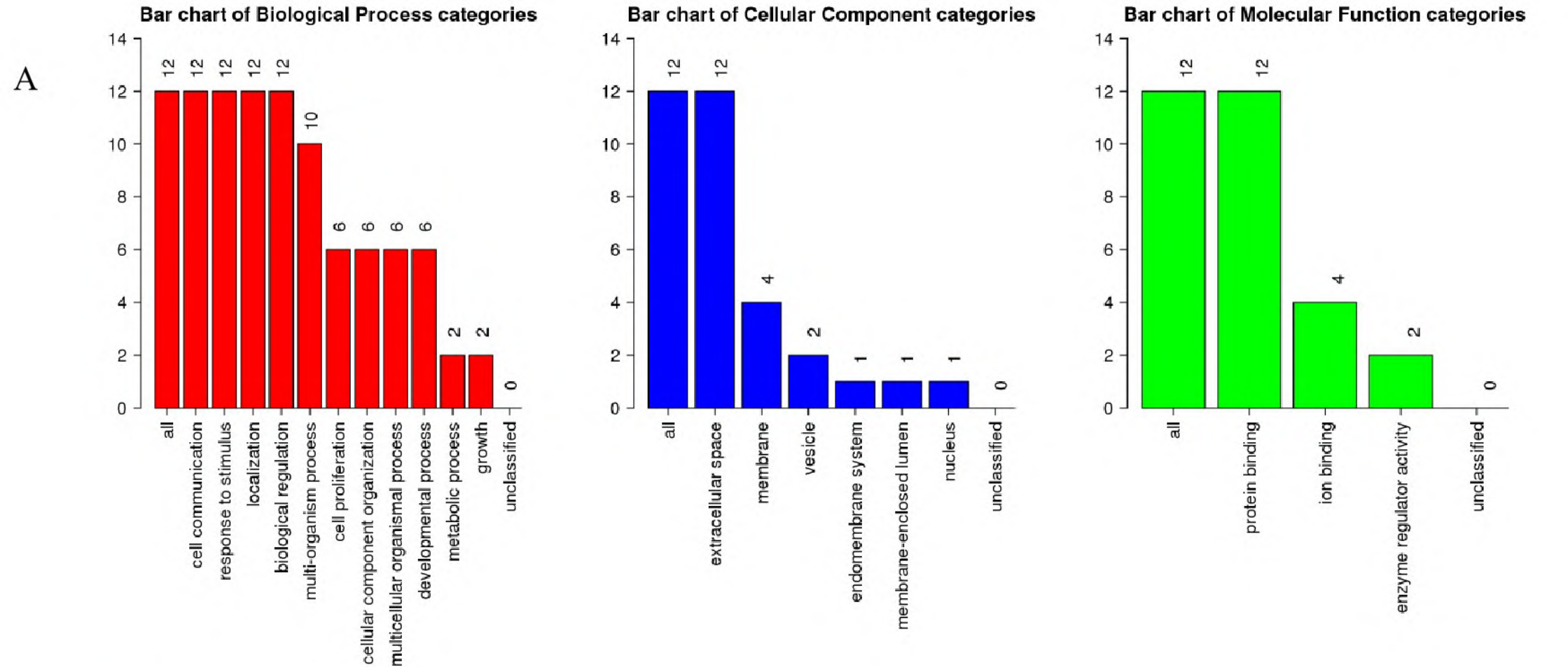

■ FDR  $\leq 0.05$  ■ FDR  $> 0.05$

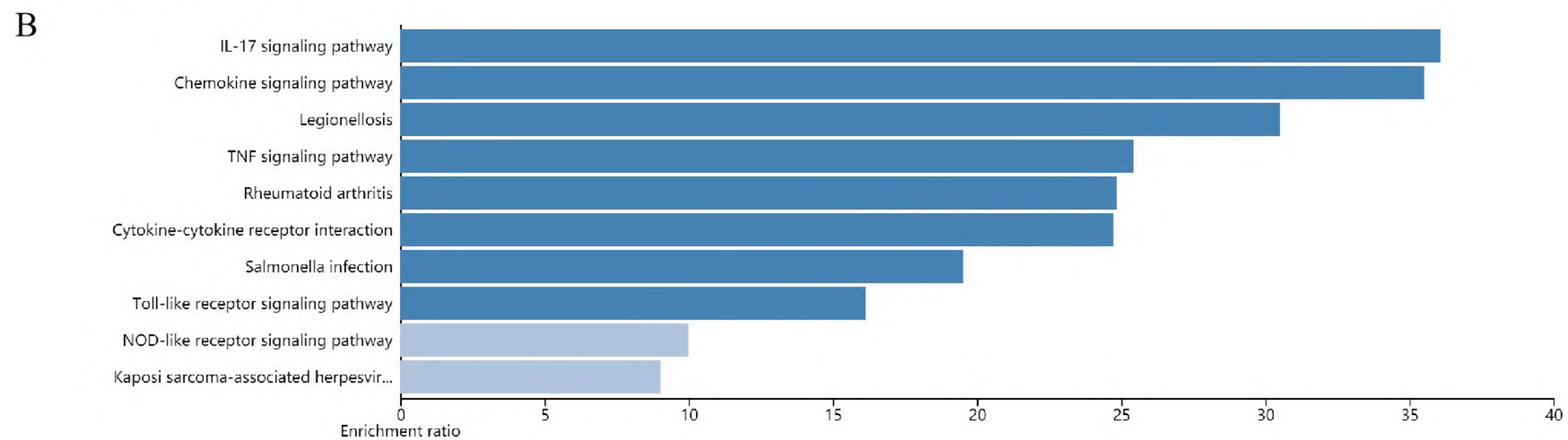

Supplement: Supplementary 3 — Supplementary Figure 3: Gene Ontology (GO) and Kyoto Encyclopedia of Genes and Genomes (KEGG) analyses of the CXCLs. (A) Bar charts of GO analysis. (B) Bar charts of KEGG pathway analysis. [file 5223657.f3.pdf]

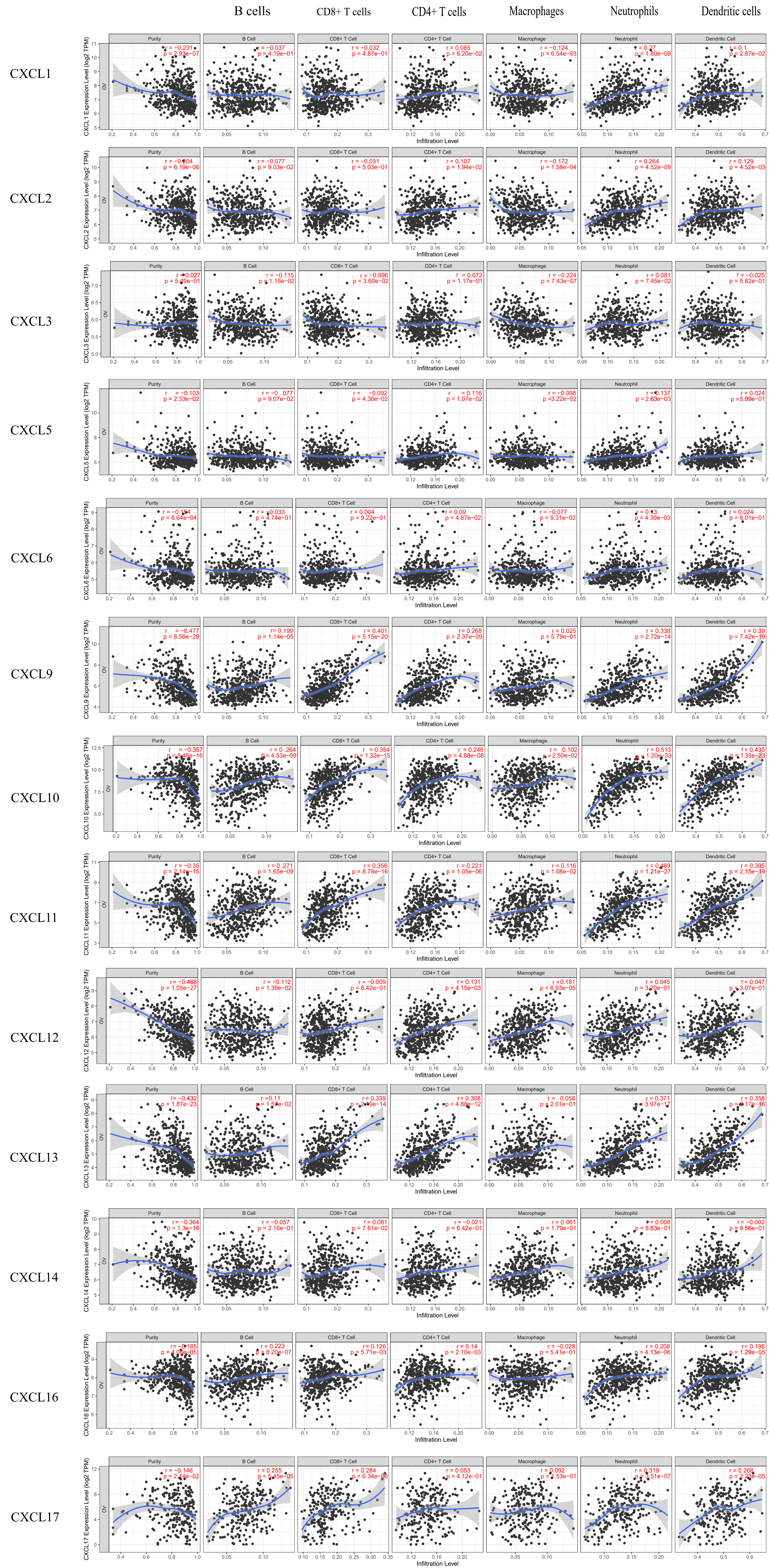

Supplement: Supplementary 4 — Supplementary Figure 4: Correlation analysis of CXCLs and infiltrating immune cells. The results showed that the expression of CXCL1 was positively associated with the infiltration of macrophages (R = 0.124, P = 6.54e − 03), neutrophils (R = 0.27, P = 1.80e − 09), and dendritic cells (R = 0.10, P = 2.87e − 02). CXCL2 expression was positively associated with CD4+ T cell (R = 0.107, P = 1.94e − 2), neutrophil (R = 0.264, P = 4.52e − 09), and dendritic cell infiltration (R = 0.129, P = 4.52e − 03) but negatively associated with macrophage infiltration (R = −0.172, P = 1.58e − 04; Figure 6(b)). CXCL3 expression was negatively associated with CD8+ T cells (R = 0.096, P = 1.16e − 2), B cells (R = −0.115, P = 1.16e − 02), and macrophage infiltration (R = −0.224, P = 7.43e − 07). CXCL5 expression was negatively associated with CD8+ T cells (R = −0.092, P = 4.36e − 2) and macrophage infiltration (R = −0.098, P = 3.22e − 02) but was significantly positively correlated with CD4+ T cells (R = 0.116, P = 1.07e − 2) and neutrophils (R = −0.137, P = 2.63e − 3). CXCL6 expression was positively correlated with CD4+T cells (R = 0.09, P = 4.87e − 2) and neutrophils (R = −0.13, P = 4.39e − 3). CXCL9 expression was positively correlated with all immune cell types except macrophages (B cells, CD8+ T cells, CD4+ T cells, neutrophils, and dendritic cells; all P < 0.05). The expression of CXCL10 and CXCL11 was positively correlated with the infiltration of B cells, CD8+ T cells, CD4+ T cells, macrophages, neutrophils, and dendritic cells (all P < 0.05). CXCL12 expression was positively correlated with infiltration of CD4+ T cells (R = 0.131, P = 4.15e − 3) and macrophages (R = 0.181, P = 6.93e − 5) but negatively correlated with infiltration of B cells (R = −0.112, P = 1.39e − 2). The expression of CXCL13 and CXCL16 was positively correlated with other immune cells except macrophages. The expression of CXCL14 was not significantly associated with the infiltration of all six immune cells. C [file 5223657.f4.pdf]
